# Removal of Zn(II) and Ag(I) by *Staphylococcus epidermidis* CECT 4183 and Biosynthesis of ZnO and Ag/AgCl Nanoparticles for Biocidal Applications

**DOI:** 10.3390/toxics13060478

**Published:** 2025-06-05

**Authors:** Antonio Jesús Muñoz, Celia Martín, Francisco Espínola, Manuel Moya, Encarnación Ruiz

**Affiliations:** 1 Department of Chemical, Environmental and Materials Engineering, Universidad de Jaén, Campus Las Lagunillas, 23071 Jaén, Spain; amcobo@ujaen.es (A.J.M.); fespino@ujaen.es (F.E.); mmoya@ujaen.es (M.M.); eruiz@ujaen.es (E.R.); 2 Institute of Biorefineries Research (I3B), Universidad de Jaén, Campus Las Lagunillas, 23071 Jaén, Spain

**Keywords:** biosorption, green chemistry, nanoparticles, biocide tests, silver, zinc

## Abstract

The contamination of natural waters with heavy metals is a global problem. Biosorption is an environmentally friendly and effective technology that offers advantages when metals are present in low concentrations. It also facilitates the recovery and conversion of metals, which are valuable resources. The removal capacity of Ag(I) and Zn(II) ions by *Staphylococcus epidermidis* CECT 4183 and the ability of its cell extract to synthesize Ag/AgCl and ZnO nanoparticles were investigated. Their biocidal capacity was evaluated. The factors involved were optimized and the mechanisms were studied. The optimal conditions for Ag(I) biosorption were pH 4.5 and a biomass dose of 0.8 g/L. For Zn(II), the biomass dose was 0.2 g/L and pH 4.2. A maximum biosorption capacity (Langmuir model) of 47.43 and 65.08 mg/g, respectively, was obtained. The cell extract promoted the synthesis of Ag/AgCl and ZnO nanoparticles with average sizes below 35 nm. The ZnO nanoparticles exhibited excellent inhibitory properties against planktonic cells of five microbial strains, with MIC values ranging from 62.5 to 250 µg/mL. Their response to biofilms remained between 70% and 100% inhibition at low concentrations (125 µg/mL). The studied bacteria show potential to remove heavy metals and promote the environmentally friendly synthesis of biocidal nanoparticles.

## 1. Introduction

Water is an essential resource for life and its quality is directly linked to the balance of ecosystems. With the increase in population and industrial growth, this resource has been overexploited and its pollution has reached worrying levels [1]. In recent decades, many studies have focused on emerging pollutants represented by pharmaceutical products, but also on heavy metals, which are present at very low concentrations and, due to their nondegradable nature, persist in various environmental matrices and tend to biomagnify, entering the food chain [2]. Heavy metals are particularly harmful in aquatic ecosystems due to their mobility, stability, toxicity and bioaccumulation [3,4]. Zinc is one of the most commonly discharged metals in wastewater, and although it is an essential micronutrient, it is highly toxic as its concentration increases [5]. The main sources of emissions of this metal are the electroplating industry [6] and mining [7], which facilitate bioaccumulation processes in aquatic species that are then consumed by humans. On the other hand, silver is an increasingly used metal and one of the most toxic metals for organisms in general. It is of great environmental concern due to its toxicity to aquatic microorganisms, with sublethal effects on reproduction and early life stages of invertebrates [8]. It is also a toxic metal to humans and other animals that can be ingested through food or water. Major sources include rock erosion and industrial activities such as mining, electroplating, and the manufacture of catalytic converters, medical devices, and batteries [9,10]. The next stage of this research will focus on using real industrial wastewater. Preliminary analyses of this work have identified small concentrations of Ag(I) ions in the microgram range, confirming the importance of studying this metal.

There are several conventional physicochemical techniques for removing metal ions from contaminated streams [11]. These techniques produce concentrated sludge and have high costs, but they have high efficiencies when metal ion concentrations are high [12]. In practice, however, many metals occur at low concentrations in natural waters, making their application useless and expensive. The biosorption of heavy metals through the use of microbial biomass is presented as a versatile, ecological and cheap tool that also involves biochemical mechanisms capable of promoting the synthesis of metallic nanoparticles that can be reused in other areas. Microbial biomass offers great versatility because it has many mechanisms of interaction with metal ions, and thus many possibilities for retention and transformation. These mechanisms can occur at the cell membrane, in the periplasmic space and in the cytoplasm, but also from the cellular extract released by microorganisms. These mechanisms include (1) chemisorption, in which the metal is chemically bound to form a new chemical species, (2) physisorption, mediated by electrostatic forces, (3) biomineralization, in which the metal is expelled to the outside of the cytoplasm and precipitates in the form of sulfide, hydroxide, or carbonate, and (4) complexation, in which there is heterogeneous accumulation of metal cations from an initial precipitate mediated by electrostatic forces. These mechanisms also include the possibility of reducing metal ions by the action of functional groups, producing nanometric species, in some cases of crystalline character, which can be recovered and reused. In general, the possibility of metal recovery is another strong point of biosorption, since when applied in continuous systems from supported microbial biomass, metals can be easily recovered with an acid cycle [13]. In parallel, and as mentioned above, some of the mechanisms involved in biosorption promote the formation of nanoparticles, which means that this metal bioremediation technology can also be applied to the synthesis of nanoparticles from the metals to be removed. This is the case of zinc, copper or silver, which can give rise to nanoparticles with important applications in other technological fields. In general, the biological synthesis of nanoparticles is based on obtaining cell and plant extracts. In both cases, the extracts act as precursors for the reduction reaction that generally controls the process. The protein components present in the extracts also play a stabilizing role, favoring the stability and dispersion of the synthesized nanoparticles. Green synthesis of nanomaterials is becoming increasingly important in various scientific and technological fields due to their unique properties and wide range of applications. Zinc oxide nanoparticles (ZnO-NPs) can be used in the cosmetic, agro-food and biomedical industries. Their biomedical applications are usually based on their potent biocidal properties against pathogenic microorganisms and their low toxicity to eukaryotic cells when used at low concentrations. In addition, they can be used together with different antibiotics, seeking synergistic combinations that allow reducing the concentration of both biocidal agents, thereby reducing the risk of toxicity. The biocidal applications of ZnO-NPs are under development and offer good future prospects not only for their application in the biomedical field, but also in the agro-food field applied to surface coatings. ZnO-NPs act by releasing Zn(II) ions and generating reactive oxygen species (ROS), both of which can cause direct damage to cellular structure or combine with enzymes essential for cellular metabolism. They are also excellent photocatalysts and exhibit semiconductor properties that allow their application in the design of biosensors [14]. Silver nanoparticles (Ag-NPs) are the most widely used due to their antimicrobial, antifungal and larvicidal properties, making their use in biomedical and agricultural applications highly valued and in constant development [15]. Regarding the biocidal effect of Ag-NPs, previous studies have described different mechanisms in which the small size of these nanoparticles has a major influence, some examples are (1) direct action on the cell wall, damaging it and facilitating access to the cell interior, (2) leakage of essential cellular components due to cell damage, (3) once in the cytoplasm, NPs can interact with essential molecules containing S or P, such as DNA itself, hindering replication, (4) affecting the balance between antioxidant enzymes (GPx and CAT), inducing the formation of ROS causing oxidative stress, and (5) affecting DNA sequences involved in protein synthesis, biofilm formation or pathogenic activity [16]. Ag-NPs also have important applications in optoelectronics, sensor development, and electrical and electronic applications.

**Scheme 1:**
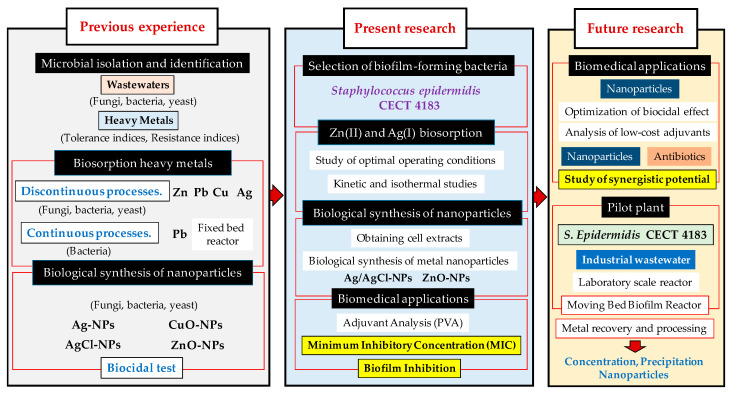
The structure of the work developed in this research is presented, along with its general context. It also shows the background and ultimate objective of the knowledge generated during the first two stages.

The aim of this study was to identify microorganisms with biotechnological potential in the field of heavy metal removal and the ability to promote their recovery and transformation into nanomaterials with potential applications in biomedicine. The authors have experience in this field of technology and some of their previous work is cited throughout the manuscript. The research is part of a broader project whose ultimate goal is to create a comprehensive repository of microorganisms with potential application in the development of biofilters for the removal, recovery and transformation of metals present in low concentrations in industrial or urban effluents. The general structure of this work and the context in which it is framed are described in Scheme 1. All this is in line with the current environmental guidelines that describe the importance of implementing in the near future specific tertiary and quaternary systems in wastewater treatment plants. To this end, the biosorption capacity of a Gram-positive bacterium, *Staphylococcus epidermidis* CECT 4183, against Ag(I) and Zn(II) ions was evaluated. Its ability to synthesize Ag/AgCl nanoparticles and ZnO nanoparticles from its cell extract was also studied. Finally, the biocidal properties of these nanoparticles against planktonic cells and microbial biofilms of different microorganisms were determined.

## 2. Materials and Methods

### 2.1. Biomass Preparation

A Gram-positive coccus was selected that was small in size and had demonstrated the ability to induce biofilm formation. This trait made it a candidate for application in a future phase of research focused on the design of biomass supported moving bed reactors. A pre-inoculum of the bacterium *Staphylococcus epidermidis* CECT 4183 was grown in liquid medium No. 1 (beef extract 5 g, peptone 10 g, NaCl 5 g, distilled water 1 L) for 24 h, at 150 rpm and 27 °C in a BIOBASE thermostatic orbital model BJPX-N (Jinan, China). From the pre-inoculum, 10 flasks of 250 mL each were inoculated with 200 mL of medium No. 1. The flasks were kept under the same conditions for 24 h. The biomass was then washed repeatedly with 0.1 M NaNO_3_ prepared in sterile distilled water. A concentrated cell suspension was obtained from which different aliquots were taken and kept at 104 °C for 24 h to calculate the dry weight, which allowed the cell concentration of the suspension to be accurately determined. Meanwhile, the mother suspension was kept at 4 °C. In parallel, the biomass for obtaining the cell extract that would later be used in the nanoparticle synthesis tests was prepared in the same way.

### 2.2. Biosorption Test

For the biosorption tests, two types of metallic solutions of two different salts were used: AgNO_3_ and ZnSO_4_·7H_2_O. In both cases and for all the biosorption tests, the fresh biomass obtained (see biomass preparation section) was contacted with the metallic solutions in 100 mL Enlermeyer flasks with a working volume of 50 mL. The tests were carried out in duplicate at 27 °C and maintained at 200 rpm in the orbit described in the previous section. In all cases, samples were taken before and after the biosorption process and filtered with 0.22 µm PES filters. The samples were analyzed in a Perkin Elmer AAnalyst 800 instrument (Midland, ON, Canada), which allowed the determination of the residual metal concentration (*C_f_*, mg/L). The biosorption capacity (*q*, mg/g) in each case was obtained by applying Equation (1), where *C_i_* is the initial concentration (mg/L), *V* is the volume expressed in L and *m* is the dry biomass (g).

(1) $$q=\frac{\left(C_{i}-C_{f}\right)V}{m}$$

A rotatable central composite design (RCCD), with five levels per factor and five center points, and response surface methodology (RSM) were used to identify the experimental conditions that produced the best biosorption responses for Ag(I) and Zn(II) ions. For silver, a range between 4.5 and 7 for pH and between 0.3 and 0.8 g/L for biomass dose was investigated. For zinc, the ranges were 4.2–6.2 and 0.2–0.8 g/L for pH and biomass dose, respectively.

Kinetic tests were performed only for zinc, as previous studies have shown that this metal usually presents two distinct phases during the biosorption process, the first corresponding to rapid adsorption and the second involving bioaccumulation mechanisms that last longer [17]. The tests were carried out in duplicate, using the optimal conditions obtained in the experimental design and an initial metal concentration of 50 mg/L. The contact time was extended to 6 days under the conditions described above. The experimental results were fitted to two kinetic models. Equation (2) corresponds to a pseudo first-order model, the Lagergren model [18], and Equation (3) corresponds to a pseudo second-order model, the Ho model [19].

(2) $$q=q_{e}(1-e^{-kt})$$

(3) $$q=\frac{q_{e}}{\frac{1}{ktq_{e}}+1}$$

In the equations, *q* is the biosorption capacity expressed in mg of metal per gram of dry biomass for any given time (*t*, min), *q_e_* is the same value under equilibrium conditions, and *k* is the kinetic constant for each case expressed in min^−1^ (Lagergren model) and g/mg·min (Ho model).

The equilibrium tests were carried out at different metal concentrations. For zinc, concentrations between 13 and 115 mg/L and a contact time determined by the results of the kinetic study were chosen. For silver, the range between 20 and 310 mg/L and a contact time of 4 days were used. For the two metals studied, the experimental data obtained in the equilibrium tests were fitted to the Langmuir [20], Freundlich [21], Sips [22] and Redlich–Peterson [23] models. The equations and parameters are described in Table 1.

### 2.3. Biomass Characterization

To analyze the mechanisms involved in metal biosorption, biomass samples were taken before and after the process and treated according to the technique used. Fourier transform infrared spectroscopy (FT-IR) and X-ray diffraction (XRD) require the sample to be in the solid state and without moisture. In these two cases, the samples were dried at 60 °C until a stable weight was obtained, ensuring the total absence of moisture, and then analyzed. In the case of scanning electron microscopy with dark field emission and X-ray scattering detector (FESEM-EDX), the samples were prepared as described by Muñoz et al. [24].

To identify the involvement of functional groups in the retention of Ag(I) and Zn(II) ions, a VERTEX 70 FT-IR instrument (Bruker Corporation, Billerica, MA, USA) was used in the range between 400 and 4000 cm^−1^. For the identification of crystalline metallic species formed during the biosorption stage, the samples were subjected to XRD analysis in a Malvern Panalytical Empyrean instrument (Malvern, UK). Finally, the samples were subjected to FESEM-EDX analysis to detect and characterize the presence of metallic precipitates on the surface of microbial cells, using a MERLIN Carl Zeiss instrument (Göttingen, Germany).

### 2.4. Synthesis and Characterization of Nanoparticles

To investigate the ability of *S. epidermidis* to promote the synthesis of ZnO and Ag nanoparticles, the cellular extract of the microorganism was obtained as described by Muñoz et al. [25]. For the synthesis of ZnO-NPs, several preliminary tests were carried out to optimize the volume of reagents that gave the best performance values. Finally, 125 mL of the cellular extract was brought into contact, drop by drop, with 500 mL of a zinc acetate solution (ZnC_4_H_6_O_4_·2H_2_O) maintained at 40 °C with constant stirring. The mixture was kept under these conditions for 1 h. The precipitate was then collected and washed four times with ultrapure water in cycles of 5500 rpm/4 min. The concentrate obtained was kept at 60 °C for 48 h and then ground in a ceramic mortar. Finally, the powder obtained was calcined at 500 °C/2 h. The powder was then ground in an agate mortar to obtain a whitish powder, presumably formed by ZnO nanoparticles (ZnO-NPs), which was finally characterized by UV-vis spectrophotometry, FT-IR, XRD, FESEM-EDX and transmission electron microscopy (TEM) in a JEOL model JEM-1010 instrument (Jeol Company, Peabody, MA, USA).

For the synthesis of silver nanoparticles (Ag/AgCl-NPs), the same cell extract was contacted with a 10 mM AgNO_3_ solution at a ratio of 180 mL extract to 20 mL metal solution. The mixture was kept at 200 rpm and 27 °C for 14 days and measured by UV-visible spectrophotometry (UV-vis) between 200 and 800 nm every 24 h to control the synthesis process. Finally, it was washed with ultrapure water and centrifuged at 11,000 rpm in a Hettich model Rotina 420-R apparatus (Beverly, MA, USA) to obtain a colloidal suspension of silver nanoparticles (Ag/AgCl-NPs).

From the spectra obtained by XRD, a first approximation of the average crystal size of the nanoparticles (*d*, nm) was made using the Debye–Scherrer equation (Equation (4)):

(4) $$d=\frac{k\lambda }{\beta cos\theta }$$

where *k* has a constant value of 0.9, *λ* is the wavelength (nm) of the incident X-rays, *β* is the width of the half-peak height of the spectrum expressed in radians, and *θ* is the Bragg diffraction angle.

### 2.5. Study of the Biocidal Capacity of Nanoparticles: Planktonic Cells vs. Biofilms

In order to study the biocidal capacity of the nanoparticles obtained, biocidal tests were carried out against five different microorganisms that had been studied in previous stages of the research [25,26], for comparative purposes. These were the Gram-negative bacteria *Escherichia coli* (CECT 101) and *Pseudomonas fluorescens* (CECT 378), the Gram-positive bacteria *Bacillus cereus* (CECT 131) and *Staphylococcus epidermidis* (CECT 4183), and the yeast *Rhodotorula mucilaginosa* 1S1 (CECT 13212), isolated from urban wastewater by the research group [27]. The biocidal potential was studied by determining the minimum inhibitory concentration (MIC) of the obtained nanoparticles and also their ability to inhibit the formation of microbial biofilms, obtaining the minimum biofilm inhibitory concentration (MBIC) [28].

To obtain the MIC, two types of tests were carried out, as described in Muñoz et al. [25], using the same equipment described in that work. In one of them, an adjuvant (polyvinyl alcohol, PVA) was incorporated to improve the dispersion of the nanoparticles, with the aim of improving their biocidal response. At the same time, a specific protocol was designed to determine the MBIC, based on the biocidal tests carried out in 96-well microplates described in the aforementioned work. However, in these tests, Na_2_SiO_3_ (10%) was used as a deflocculant of the colloidal suspension of nanoparticles [29]. Under these conditions, after inoculating the microorganisms in triplicate, the microplates were incubated for 72 h at 27 °C and then the absorbance was read at 630 nm in a TECAN Infinite M Plex microplate reader (Männedorf, Switzerland). The wells were then washed with large volumes of sterile distilled water, shaken upside down each time to remove any planktonic cells. A volume of 270 µL of 1% crystal violet solution was then pipetted in for 10 min before washing again. To avoid the presence of traces of dye in microdroplets adhering to the plastic, several immersion steps in sterile water were included, which was changed each time the procedure was repeated. Finally, 270 µL of 95% ethanol was pipetted in for 15 min at 5 °C to avoid excessive evaporation. The plates were then transferred to the microplate reader and the absorbance at 540 nm was measured, providing an indirect quantitative measure of the amount of biofilm formed during the 72 h of the experiment. The results were expressed as percentage inhibition (% inhibition) in relation to the positive control for each concentration tested (Equation (5)) and in µg/mL to express MBIC.

(5) $$\%\;Inhibition=100-\frac{100\times DO_{c}}{DO_{f}}$$

where *DO_c_* is the wavelength value for the maximum growth value in the positive control and *DO_f_* is the same for the maximum growth value at the different toxic concentrations.

## 3. Results and Discussion

### 3.1. Experimental Design

**Figure 1:**
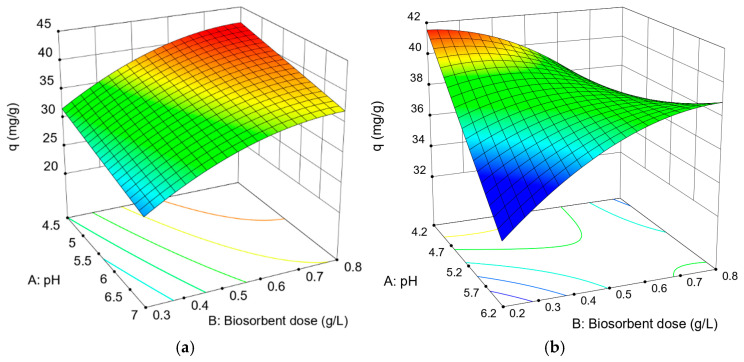
(**a**): Response surface plot for Ag(I) biosorption by *S. epidermidis* CECT 4183; (**b**): the same for the case of Zn(II) biosorption.

Figure 1a,b show the response surfaces obtained for the biosorption of Ag(I) and Zn(II), respectively. These response surfaces were obtained after fitting the experimental data (Tables S1 and S2) to a quadratic model represented by Equations (6) (for Ag) and (7) (for Zn). The equations are expressed in real values and the factors represented are biomass dose (*B*) expressed in g/L and pH (*A*), while the dependent variable (*q_e_*) is expressed in mg of metal retained per g of dry biomass.

(6) $$q_{e}=25.04-2.07A+65.35B-40.30B^{2}\pm 0.72$$

(7) $$q_{e}=69.94-6.64A-39.01B+10.69AB-16.68B^{2}\pm 0.62$$

The models fit the experimental data well, as indicated by the statistical parameters of the fit (Tables S4 and S5). As shown in Figure 1a, the pH exerts a negative influence on the silver biosorption process, although not very significant; the influence of the biosorbent dose factor is greater and positive, and presents a small curvature. For zinc biosorption the pH still exerts a linear influence and the biosorbent dosage presents a small curvature but, in this process, the most influential term is the interaction between the factors. As shown in Figure 1b, the surface rotates according to whether the lower or upper range of each factor is considered, so that the influence of each factor depends on the value of the other factor.

As can be deduced from Equations (6) and (7) and observed in Figure 1a,b, the optimum operating conditions of the biosorption process are, for silver: biomass dose 0.8 g/L and pH 4.5, and for zinc: biomass dose 0.2 g/L and pH 4.2.

### 3.2. Kinetic and Equilibrium Studies: Biosorption Isotherms

The biosorption kinetics tests were performed specifically with zinc and showed similar values for the fit of the experimental data with the two models used (Table S3). The best fit was obtained with the pseudo-first order model with an R^2^ of 0.94, while the pseudo-second order model showed an R^2^ of 0.92. These data seem to indicate that the limiting step in the biosorption process was the mass transfer of the metal to the surface, so the nature of the adsorbate played a major role in the process. However, the pseudo-second order fit was also not bad, which could indicate that, in addition to chemisorption phenomena, complexation and bioaccumulation phenomena could be involved. The above is consistent with what was observed in this work.

Table 1 shows the values obtained for the different parameters used to fit the experimental data to the equilibrium models. The model that best fitted the data for the case of Ag(I) biosorption was the Sips model with an R^2^ of 0.93. On the other hand, the model that best fitted the equilibrium data for the case of Zn(II) biosorption was the Redlich–Peterson model with an R^2^ of 0.97. However, in both cases, all the models fitted the experimental data obtained well.

**Figure 2:**
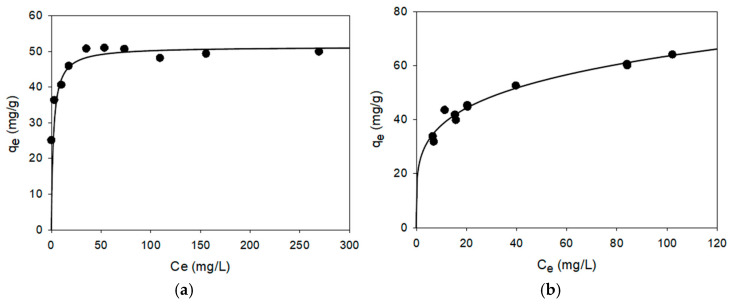
Isotherms obtained for the biosorption of Ag(I) and Zn(II) by *Staphylococcus epidermidis* CECT 4183. (**a**) Langmuir isotherm for the biosorption of silver. (**b**) Freundlich isotherm for the biosorption of zinc.

Figure 2a shows the Langmuir isotherm for the case of Ag(I) biosorption, which gave a *q_m_* of 47.43 mg/g. This theoretical value was very close to the maximum value obtained experimentally. There are not many works on silver biosorption with Gram-positive bacteria, but the *q_m_* obtained by *S. epidermidis* is among the best reported for this category. Some authors have found high adsorption values (91.7 mg/g) for a Gram-positive bacterium, but it was a bacillus, *Bacillus cereus* [30]. On the other hand, Wang et al. found discrete values of 13.43 mg/g for the Gram-negative bacterium *Magnetospirillum gryphiswaldense* [31]. Other authors have worked with macrofungi and obtained results similar to those in this work, with *q_m_* values between 39.84 and 45.45 mg/g for the Langmuir model [32].

In parallel, Figure 2b shows the Freundlich isotherm obtained for the biosorption of Zn(II). *S. epidermidis* provided a *q_m_* of 65.08 mg/g for the Langmuir model with a good fit to the experimental data (R^2^ = 0.93). This value is among the best for Gram-positive bacteria and also in general. Other authors working with Gram-negative bacteria found lower values, with a *q_m_* of 46.1 and 36 mg/g for *Pseudomonas aeruginosa* [33] and *Acinetobacter* sp. [34], respectively. Li et al. investigated the ability of a Gram-positive bacterium of the genus *Streptomyces* to resist heavy metals; bacteria of this genus also have the ability to form vegetative hyphae. The authors worked with live and dead cells and found *q_m_* values of 42.75 and 54 mg/g, respectively [35]. Based on the above, it can be concluded that the *S. epidermidis* bacteria have good characteristics for the elimination of heavy metals. Table 2 shows the data obtained by other authors for different types of biomass under conditions similar to those used in this work and as can be seen, the values obtained are among the best.

### 3.3. Biosorption Mechanisms

**Figure 3:**
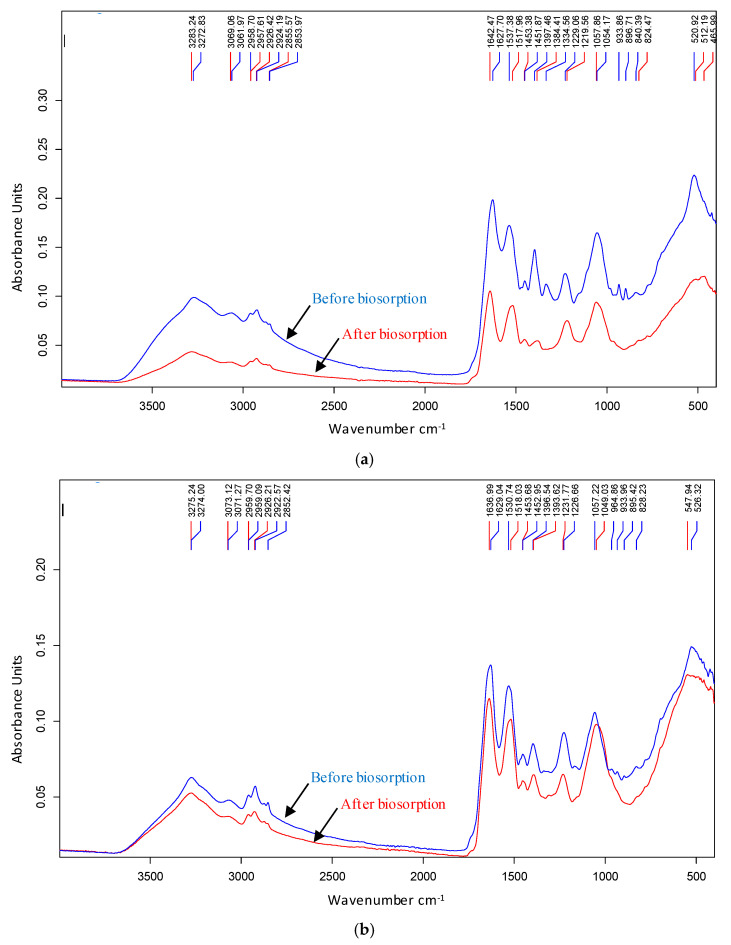
FT-IR spectra obtained before and after the biosorption process of silver (**a**) and zinc (**b**) for *S. epidermidis* CECT 4183.

Figure 3 shows the FT-IR spectra obtained before and after the biosorption stage of Ag(I) and Zn(II). The spectra show shifts in some characteristic bands and a significant loss of intensity after the biosorption stage, revealing the involvement of numerous functional groups in the process. A greater loss of intensity is observed in the case of silver, indicating a greater involvement of functional groups in the retention process of this metal.

Table 3 shows the bands affected and the shifts caused during this stage. In the case of silver (Figure 3a), changes were observed that mainly affected the bands associated with amino, hydroxyl, methyl, methyl and carbonyl groups [36], whereas in the case of zinc (Figure 3b), the involvement of methyl, methylene, carbonyl and phosphate groups was observed [37]. Therefore, in both cases a strong involvement of the functional groups in the biosorption process was observed, indicating that surface phenomena are involved in the process.

**Figure 4:**
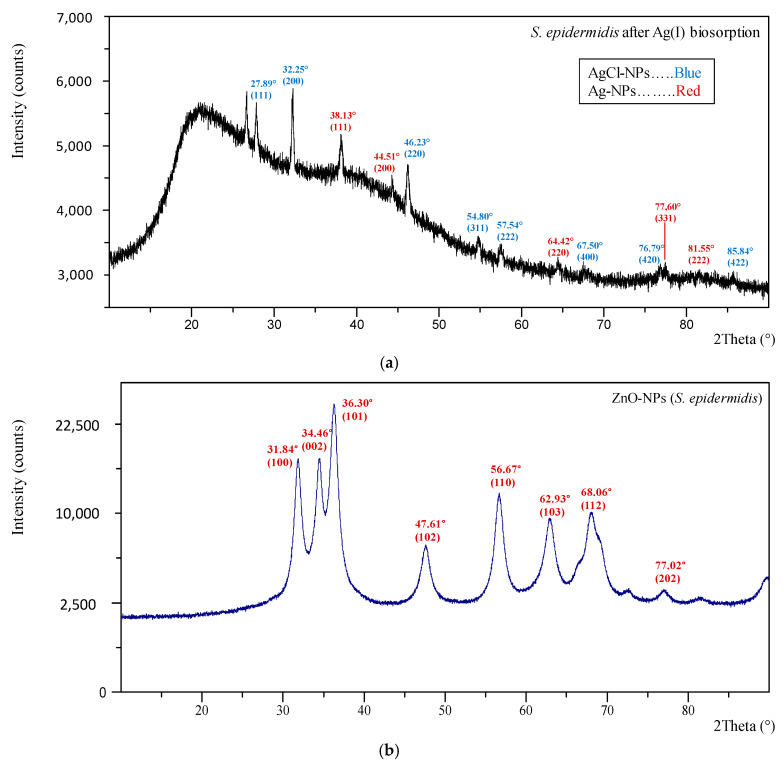
(**a**): XRD spectra obtained for *S. epidermidis* CECT 4183 after the Ag(I) biosorption step, identifying the characteristic bands and the corresponding Miller indices of the Ag/AgCl NPs synthesized in the process. (**b**): XRD spectra obtained after the biological synthesis step of ZnO NPs from the cell extract of *S. epidermidis*, showing the characteristic bands together with the Miller indices.

In parallel, the samples were also analyzed by XRD to identify the presence of crystalline metallic species compatible with the presence of nanoparticles formed during the biosorption of metals. Figure 4a shows the spectrum obtained for the case of silver, and it can be observed that the biomass analyzed after the Ag(I) biosorption step presented peak characteristics of AgCl and Ag nanoparticles [38,39], indicating that *S. epidermidis* promotes the synthesis of nanoparticles during silver biosorption. Although the XRD peaks of AgCl and AgCl-NPs coincide, when the Debye–Scherrer equation was applied, nanometric values were obtained, unlike what would occur with AgCl crystals (micrometer order), showing that the detected peaks were due to AgCl-NPs. At the same time, the spectra obtained by UV-vis analysis (Section 3.4) during the synthesis phase also revealed the presence of this type of nanoparticles, supporting the hypothesis that they were also formed during the biosorption process. Li et al. found that another Gram-positive bacterium, *Lactobacillus* sp. A09, had the ability to reduce Ag(I) to Ag(0) because the groups present in the polysaccharides acted as electron donors during silver biosorption, and suggested that this fact might provide an opportunity for the synthesis of Ag-NPs [40]. When the highest intensity peaks were analyzed using the Debye–Scherrer equation, very small crystal diameters with a face-centered cubic (FCC) structure were obtained, with values of 34 and 23 nm, respectively. In contrast, the spectrum obtained after the Zn(II) biosorption step did not identify any peaks compatible with the presence of ZnO-NPs, indicating that nanoparticles were not formed during this step or, if they were, these crystalline species were present at very low concentrations.

**Figure 5:**
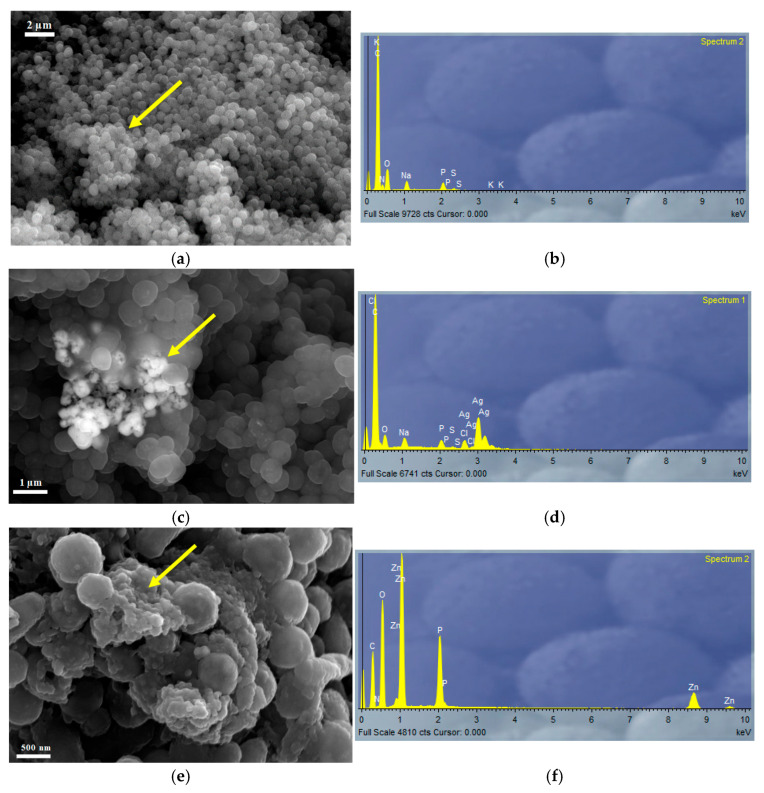
FESEM images obtained before (**a**) and after the biosorption process of Ag(I) and Zn(II) ions by *S. epidermidis* CECT 4183 ((**c**) and (**e**), respectively). EDX spectra obtained before (**b**) and after biosorption of Ag(I) (**d**) and Zn(II) (**f**).

Finally, FESEM-EDX analysis was performed. Figure 5 shows the images obtained before and after the biosorption process. Figure 5a shows an image obtained before the biosorption process, while Figure 5c,e show the images obtained after the biosorption of Ag(I) and Zn(II), respectively. In the case of zinc, the images show numerous precipitates that form complexes on the cell surface, which could involve the participation of phosphate, amino and carboxyl groups [41], which were revealed in the FT-IR analysis. In support of this hypothesis, the EDX spectrum (Figure 5f) shows a pronounced phosphorus peak that does not appear in the spectra of the original biomass, indicating that this element is concentrated after the biosorption of Zn(II). In the case of silver, microprecipitates were observed distributed throughout the cells, which could correspond to the Ag/AgCl NPs identified in the XRD analysis and which could be promoted by chemical reduction phenomena of the hydroxyl groups whose participation was detected in the FT-IR analysis. The presence of characteristic Cl peaks in the EDX spectrum would also support this assertion. In parallel, Figure 5c shows the presence of complexation phenomena distributed throughout the sample. All this suggests that chemical reduction phenomena, surface biosorption, microprecipitation and complexation may be involved in the biosorption of Ag(I) by *S. epidermidis* CECT 4183. Other authors have come to similar conclusions. Chen et al. identified surface sorption as the main mechanism through covalent bonds with different atoms (O, S and P), electrostatic interactions and microprecipitation [42].

### 3.4. Characterization of Nanoparticles

**Figure 6:**
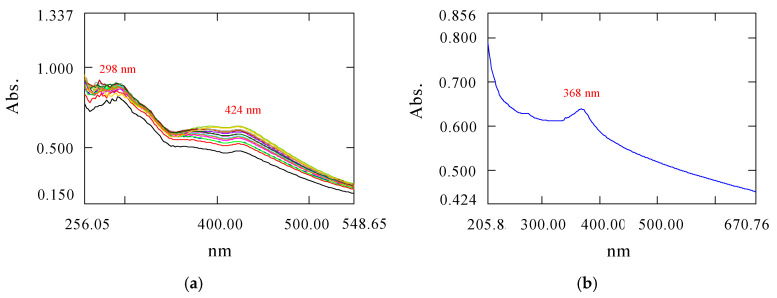
UV-vis spectra obtained during the days (each day corresponds to a color) that the Ag/AgCl nanoparticle synthesis process lasted (**a**) and after this process in the case of ZnO-NPs (**b**). The graphs show the characteristic wavelengths of each type of nanoparticle.

During and after the nanoparticle synthesis tests, various characterization techniques were used to monitor the process and confirm its successful completion. Figure 6 shows the UV-vis spectra obtained for both types of nanoparticles. Figure 6 shows the evolution of the Ag/AgCl-NPs synthesis over 20 days and shows two characteristic peaks at 298 nm and 424 nm, which are related to the plasmonic resonance of the AgCl and Ag nanoparticles, respectively [43]. Although the results indicated that the *S. epidermidis* cell extract has the potential to synthesize Ag/AgCl-NPs, it was observed that the process was slow and did not provide a significant yield. On the other hand, as shown in Figure 6, when the solid obtained after the ZnO-NPs synthesis process was suspended in ultrapure water and analyzed by UV-vis, it showed a well-defined peak at 368 nm, which is associated with the presence of this type of nanoparticles and indicated that the process had been carried out successfully [44]. The yield obtained for the ZnO-NPs was significantly better than that of the Ag/AgCl-NPs.

At the same time, Figure 4b shows the XRD spectrum obtained from the solid obtained after the ZnO-NPs synthesis process. The spectrum was obtained by analyzing the data provided by the DRX equipment with the free software, Data Viewer (version 2.2). The detected peaks are identified on the spectrum along with the Miller indices and were located exactly at the characteristic angles for this type of nanoparticles. It was shown that the obtained solid was high purity ZnO-NPs as indicated by the intense and narrow peaks of the spectrum [45]. When the most intense peak was analyzed using the Debye–Scherrer equation, it was determined that the average crystal size was 9 nm, a smaller size than that obtained by other authors [46]. In principle, this would be a nanomaterial with high potential for its application as a biocidal agent, and for this reason its morphology was subsequently evaluated by FESEM and TEM analysis. Although the mechanism of formation of ZnO-NPs by green synthesis is not clear, some authors have proposed plausible models in which the Zn(II) ions of the precursor bind to hydroxyl groups to form a stable complex structure that releases the nanoparticles after the calcination step [47].

**Figure 7:**
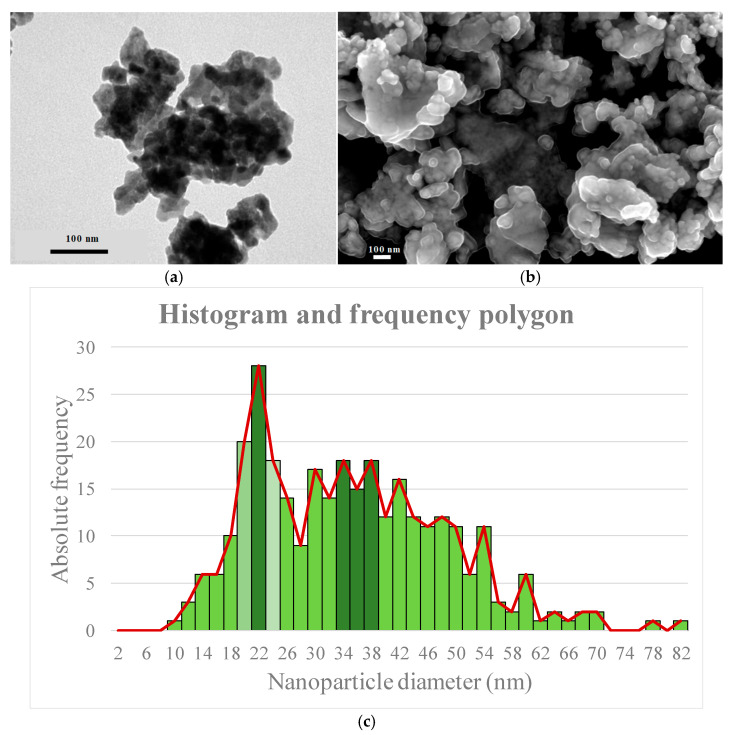
(**a**): TEM image of ZnO NPs synthesized by *S. epidermidis* CECT 4183. (**b**): FESEM image of the same type of nanoparticles. (**c**): Histogram and frequency polygon obtained from image (**b**), showing the average size distribution of the nanoparticles.

The morphological analysis is shown in Figure 7, which shows a TEM image (Figure 7a) and a FESEM image (Figure 7b). In these images, it can be observed that the morphology of the ZnO NPs is predominantly spherical and with uniform sizes. The free software ImageJ (version 1.53e) was used to estimate the average actual size, which yielded a value of 22 nm, although the histogram in Figure 7c shows considerable variability with sizes ranging from 2 to 70 nm. These data are similar to the theoretical value and, together with the spherical and uniform morphology, indicate that this type of nanoparticle is a good candidate for use in biocidal testing. This circumstance, together with the low yield in the synthesis of Ag/AgC-NPs, made ZnO-NPs the ones chosen to test their biocidal activity.

### 3.5. Biocidal Tests: Determination of MIC and MBIC

Table 4 shows the MIC results obtained in the biocidal tests performed with the ZnO NPs. As can be seen, the addition of PVA significantly improved the inhibitory capacity of the nanoparticles against the microbial strains tested. For *B. cereus* and *P. fluorescens*, the addition of PVA improved the inhibitory response 8 times compared to the original obtained in solid medium. For *E. coli*, the inhibition was improved 4 times compared to the original. For *S. epidermidis*, PVA did not offer any improvement and MIC values identical to those obtained in the first protocol (62.5–125 µg/mL) were obtained, which were already very good and indicated that the bacterium was very sensitive to nanoparticles. Likewise, the yeast *R. mucilaginosa* was tested only with nanoparticles adjuvanted with PVA and showed values identical to those obtained with the previous bacteria. In general, the adjuvanted ZnO-NPs offered low MIC values between 62.5 and 250 µg/mL, indicating a good biocidal activity against the tested strains. The effectiveness of ZnO NPs as biocidal agents is justified by (1) their large surface area due to their small size and spherical morphology in this case, (2) a greater amount of oxygen vacancies since they are metal oxides that have also undergone a calcination process, (3) the dissociation and diffusion of Zn(II) ions, and (4) their role in the synthesis of reactive oxygen species (ROS) [29,48,49]. In its oxide form, Zn is sensitive to UV-vis light, which causes the electrons in its valence band to shift and generate positive charges. These charges initiate redox reactions with O_2_ and H_2_O, releasing highly reactive oxygen species (ROS) that damage microbial cells [50].

Although comparisons are complicated by the wide variety of methods used and the existence of different strains of the same species, in general the MIC values obtained in this work are similar and, in some cases, better than those obtained by other authors who synthesized ZnO-NPs using different procedures. Marques et al. optimized a method for the synthesis of ZnO-NPs by a microwave-assisted hydrothermal process. These nanoparticles exhibited good MIC values against different microbial strains. Two different strains of S. aureus and one of *S. epidermidis* showed values between 256 and 512 µg/mL for most of the nanoparticles and in some cases above these values [51]. Elankathirselvan et al. reported a MIC value of 200 µg/mL for *B. cereus*, which is a better value than that found in this work, although the method used (diffusion in agar wells) is less reliable and precise than the one used in this work, which analyzes the growth dynamics at 24 h [48]. On the other hand, in a comprehensive work, Sirelkhatim et al. reproduced the results obtained by different authors working with *S. aureus* and *E. coli*, obtaining MIC values between 1000 and 3100 µg/mL [29]. Ebadi et al. studied the biocidal capacity of ZnONPs synthesized from cyanobacterial cell extracts, obtaining MIC values of 2000, 64, and 2000 µg/mL for *E. coli* ATCC 59222, *S. aureus* ATCC 59223, and *P. aeruginosa* PAO1, respectively [52].

The ZnO-NPs obtained in this work were also tested for their ability to inhibit the growth of microbial biofilms. Three microorganisms were tested as described in the experimental section (Study of the biocidal capacity of nanoparticles: planktonic cells vs. biofilms) and the results are shown in Table 5, where the percentages of inhibition are related to the concentration of nanoparticles present in the nutrient medium. As can be seen, in all three cases a significant inhibition occurred, highlighting the case of *E. coli* bacteria and the yeast *R. mucilaginosa* with inhibitions of 100% from a concentration of 31.25 µg/mL in the first case and over 70% from 125 µg/mL for the yeast. *P. fluorescens* also showed very good values with inhibitions close to 70% from this last concentration of nanoparticles. With these data, it was possible to confirm that the MBIC at 72 h was in the range of 1000–2000 µg/mL for *R. mucilaginosa* and in the range of 15.63–31.25 µg/mL for *E. coli*. At the same time, it was found that the dose that inhibits *P. fluorescens* biofilm formation by 80% is in the range of 1000–2000 µg/mL. These values improve those obtained by some authors.

**Figure 8:**
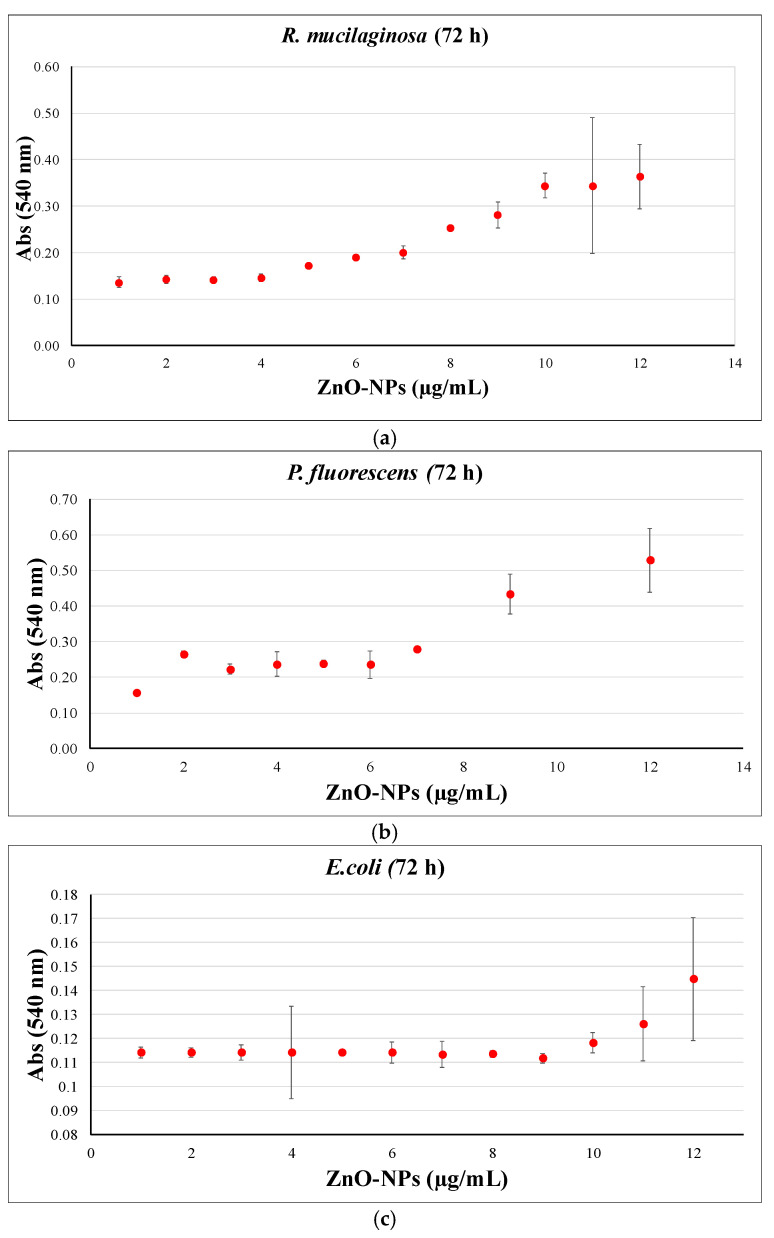
Biofilm inhibition graphs after 72 h of growth for the three microorganisms studied. The highest concentration (4000 µg/mL) is represented by the value 2 on the *x*-axis, while the lowest (7.8 µg/mL) is represented by the value 11. Values 1 and 12 were the negative and positive control respectively. Graphs (**a**–**c**) represent the inhibition values obtained for *R. mucilaginosa*, *P. fluorescens* and *E. coli*, respectively.

Asif et al. reported MBIC values of 375, 187.5, 46.8, and 93.7 μg/mL for *E. coli*, K. pneumonia, *B. cereus*, and *S. aureus*, respectively [53]. At the same time, Ebadi et al. found that the inhibition rate of different concentrations (128–2500 μg/mL) of green chemistry-synthesized ZnO-NPs ranged from 9.49 to 87.30% for *E. coli* and from 51.32 to 80% for *P. aeruginosa*, while values from 26.35 to 82.86% were obtained for *S. aureus* at concentrations from 8 to 64 μg/mL [52]. Figure 8 shows the biofilm inhibition curves as a function of ZnO-NPs concentration after 72 h of incubation. The *x*-axis represents the increasing concentration of nanoparticles, with values 1 and 12 representing the negative and positive controls, respectively. Similarly, the values between 2 and 11 represent the decreasing concentrations of nanoparticles from 4000 to 7.8 μg/mL, as shown in Table 5. The graph includes the standard deviation of the triplicates shown as error bars. The graphs clearly show that the highest concentrations inhibit biofilm formation better, while the lowest concentrations allow microbial growth more similar to the positive control in which no inhibitory toxicant was present. It can also be seen that even the lowest concentrations have a significant inhibitory effect, which in the case of *E. coli* could be considered as MBCI_90_. The biofilm inhibition results show that ZnO-NPs have great potential for use and application in clinical and food industries.

In conclusion, the positive results obtained in this work add to the available evidence for the use of ZnO-NPs in the biomedical field. Very recent studies also show that these types of nanoparticles have a very good safety profile when used with eukaryotic cells and present additional advantages when obtained by green synthesis due to their improved stability, biocompatibility and versatility in combination with antibiotics, along with their reduced adverse effects [54,55]. All this suggests that not only can they be used to protect clinical instruments or solid surfaces from colonization by pathogenic microorganisms, but that they can also be used directly or in combination with different types of antibiotics in the short term [56,57]. Table 6 summarizes the properties that other authors have identified when working with Ag/AgCl and ZnO nanoparticles, which include potent biocidal, photocatalytic, antibiofilm, anticancer, anti-inflammatory, and antioxidant activity, among others.

The good performance and future prospects of metallic nanoparticles obtained by biological synthesis from microorganisms, which, at the same time, are capable of successfully removing heavy metals from polluted wastewater, allow us to believe that the development of continuous systems that treat these effluents at source could be a reality in the short term. International pollution control regulations emphasize the need to implement tertiary and quaternary systems to remove micropollutants from wastewater. 

In this context, and knowing that many of these microorganisms are capable of forming stable microbial biofilms, as is the case with *S. epidermidis*, it is suggested that the next step in technological scaling would be the development of bioreactors that operate with biomass supported on low-cost inorganic media and that would work, in each case, with real water from the industries interested in the technology, with the aim of saving the costs derived from the controlled extraction of their waste by external companies. This transition to a laboratory pilot plant would allow for the study and definition of the parameters involved and lay the groundwork for the design of an industrial scale pilot plant. These plants could include designs that would allow for the recovery and concentration of metals removed from the effluent during the regeneration phase of the system. It is possible that a fraction of these, based on the specific characteristics of each case, could be recovered as easily purifiable metallic nanoparticles that could be applied in biomedical or other technological fields. This would not only remove dangerous inorganic pollutants from the environment, but also add value to treatment systems by turning waste into a product with enormous potential. Scheme 1 shows that, although there is still work to be done, the future prospects are good and the next steps in this research are moving in that direction.

## 4. Conclusions

The conclusions of the present work can be summarized in the following six points:

(1) *Staphylococcus epidermidis* CECT 4183 exhibited excellent characteristics for use as a biosorbent for Ag(I) and Zn(II) ions, with *q_m_* values of 47.43 and 65.08 mg/g, respectively. These values are among the best reported in scientific literature.
(2) These values were obtained under optimal operating conditions: pH 4.5 and a biomass dose of 0.8 g/L for Ag(I), and a biomass dose of 0.2 g/L and pH 4.2 for Zn(II).
(3) The cellular extract of the bacteria demonstrated good characteristics for use as a catalyst in the synthesis of Ag and ZnO nanoparticles, though its performance was significantly better when working with Zn(II) ions.
(4) High-purity ZnO-NPs were obtained, which acted as effective biocidal agents against both planktonic cells and microbial biofilms of the studied microorganisms.
(5) MIC values ranged from 62.5 to 250 µg/mL, while biofilm formation inhibitions of over 70% were achieved with exposures at low doses, as low as 125 µg/mL.
(6) In the case of *E. coli*, complete inhibition was observed with only 15.63 µg/mL, showcasing the biocidal potential of ZnO-NPs synthesized from *S. epidermidis* CECT 4183.

This indicates that *Staphylococcus epidermidis* CECT 4183 is a microorganism with significant application potential across various fields of biotechnology, ranging from heavy metal biosorption to the control of pathogenic microorganisms. These characteristics make it ideal for developing and scaling systems that continuously work with real wastewater. These systems should not only aim to prevent toxic metals present at low concentrations in industrial effluents from harming the environment, but also to recover, reuse, and/or convert the metals into final products for use in other technological areas.

## Figures and Tables

**Table 1:** Isotherm models for the biosorption equilibrium and parameters obtained for the biosorption of Ag(I) and Zn(II) by *S. epidermidis*.

Model	Equation	Parameter	Ag	Zn
Langmuir [20]	$q_{e}=\frac{q_{m}bC_{e}}{1+bC_{e}}$	q_m_	47.43	65.08
		b	8.142	0.1306
		R^2^	0.728	0.929
		(R^2^)_adj_	0.694	0.921
		Σ(q − q_cal_)^2^	175.73	83.53
Freundlich [21]	qe=KFCe1n	K_F_	34.051	22.524
		n	12.099	4.451
		R^2^	0.858	0.967
		(R^2^)_adj_	0.840	0.963
		Σ(q − q_cal_)^2^	91.61	39.42
Sips [22]	qe=KsCe1n1+asCe1n	K_s_	81.786	22.355
		a_s_	1.418	0.1969
		n	3.120	2.507
		R^2^	0.930	0.970
		(R^2^)_adj_	0.910	0.962
		Σ(q − q_cal_)^2^	45.16	35.76
Redlich–Peterson [23]	$q_{e}=\frac{K_{RP}C_{e}}{1+a_{RP}C_{e}^{\beta }}$	K_RP_	820.122	31.073
		a_RP_	21.839	1.0902
		β	0.940	0.824
		R^2^	0.902	0.971
		(R^2^)_adj_	0.873	0.963
		Σ(q − q_cal_)^2^	63.55	34.75

q_e_: biosorption capacity (mg/g) at equilibrium; q_m_: maximum biosorption capacity (mg/g); b: Langmuir biosorption equilibrium constant (L/mg); C_e_: equilibrium concentrations of metal (mg/L); K_F_: characteristic constant related to the biosorption capacity; n: characteristic constant related to the biosorption intensity; K_s_ and a_s_: Sips isotherm parameters; K_RP_, a_RP_ and β: Redlich–Peterson parameters, and β varies between 0 and 1; R^2^ is the coefficient of determination; (R^2^)_adj_ is the adjusted coefficient of determination; Σ(q − q_cal_)^2^ is the sum of the errors squared.

**Table 2:** Maximum biosorption capacity values for Ag(I) and Zn(II) obtained for different biomass types. Values obtained under comparable operating conditions are included.

Microorganism	Type	Metal	Biosorption Capacity *	Reference
*Klebsiella* sp. *3S1*	Bacteria	Zn(II)	48.4	[17]
*Pseudevernia furfuracea*	Fungi	Zn(II)	8.93	[19]
*Botryosphaeria rhodina* MAMB-05	Fungi	Ag(I)	34.67 39.23	[24]
*Rhodotorula mucilaginosa* 1S1	Yeast	Ag(I)	60.44 58.65 60.53	[26]
*Bacillus cereus*	Bacteria	Ag(I)	91.7	[30]
*Magnetospirillum gryphiswaldense*	Bacteria	Ag(I)	13.43	[31]
*Pleurotus platypus*	Macrofungus	Ag(I)	45.45 43.29 39.48	[32]
*Pseudomonas aeruginosa*	Bacteria	Zn(II)	46.1	[33]
*Acinetobacter* sp.	Bacteria	Zn(II)	36	[34]
*Streptomyces ciscaucasicus* (live) *Streptomyces ciscaucasicus* (death)	Bacteria	Zn(II)	42.75 54	[35]
*S. epidermidis CECT 4183*		Ag(I) Zn(II)	65.08 47.43	This work

(*) Maximum biosorption capacity (q_m_) expressed in mg of metal per gram of dry biomass.

**Table 3:** Location, shift and band assignments of the FT-IR spectra obtained before and after the Ag(I) and Zn(II) biosorption step. All results are expressed in cm^−1^.

Ag(I)			Zn(II)			
Before	After	Shift	Before	After	Shift	Assignment
3273	3283	10	-	-	-	Estretching vibrations of amino (-NH) and hydroxyl (-OH) group
3062	3069	7	-	-	-	Estretching C-H groups
			2852	-	-	Symmetric C-H estretching vibrations (-CH_3_)
1628	1642	14	1629	1637	8	C=C/C=C/C-N Stretching and N-H bending vibrations (Amida I)
1537	1518	19	1531	1518	13	-NO asymmetric stretching
1397	1384	13				C-H bending (-CH_2_)
-	1335		1337	1309	29	C-H symmetrical bending vibrations (-CH_3_)/O-H bending vibrations
1220	1229	9	1227	1232	-	C-O stretching,
1058	1054	4	1057	1049	8	C-O and P-O stretches
-	-	-	965	-	-	C=C flexion and P-O stretching
-	934	-	934	-	-	C=C flexion and P-O stretching
-	897	-	895	-	-	C=C flexion and P-O stretching
824	840	16	828	-	-	C=C bending
877	-	-	-	-	-	C=C bending
-	-	-	777	-	-	C-H bending
-	-	-	695	-	-	C-H bending
512	521	9	526	548	22	Possible involvement of COO, nitro and disulfide groups
466	-	-	-	-	-	Possible involvement of COO, nitro and disulfide groups

**Table 4:** Minimum inhibitory concentration (MIC) of different microorganisms against ZnO-NPs obtained from *S epidermidis* CECT 4183 before and after treating it with PVA (10%). The results are expressed in µg/mL.

Bacteria	ZnO-NPs	ZnO-NPs + PVA(10%) *
*B. cereus*	1000–2000	125–250
*S. epidermidis*	62.5–125	62.5–125
*E. coli*	250–500	62.5–125
*P. fluorescens*	1000–2000	125–250
*R. mucilaginosa 1S1*	-	62.5–125

* PVA: polyvinyl alcohol.

**Table 5:** Percentage of inhibition obtained in the microbial biofilm formation tests exposed to different concentrations of ZnO-NPs.

*Rhodotorula mucilaginosa* 1S1										
ZnO-NPs (µg/mL)	4000	2000	1000	500	250	125	62.50	31.25	15.63	7.80
Inhibition (%)	97.1	97.5	95.6	84.3	76.3	71.7	48.1	36.3	8.7	8.6
*Pseudomonas fluorescens*										
ZnO-NPs (µg/mL)	4000	2000	1000	500	250	125	62.50	31.25	15.63	7.80
Inhibition (%)	71.0	82.2	78.4	77.9	78.8	67.3	-	25.3	-	-
*Escherichia coli*										
ZnO-NPs (µg/mL)	4000	2000	1000	500	250	125	62.50	31.25	15.63	7.80
Inhibition (%)	100	100	100	100	100	100	100	100	98.9	96.8

**Table 6:** Different properties identified in Ag/AgCl and ZnO nanoparticles. The type of synthesis from which they originate and their precursors are also shown.

Precursor of Synthesis	Type	Nanopartícles	Activity	Reference
*Origanum ehrenbergii*	Plant	Ag/AgCl	Antibacterial Antibiofilm Antioxidant	[16]
*Solidago altissima*	Plant	Ag/AgCl	Antibacterial Photocatalytic Activity	[43]
*Garlic peel*	Plant residue	ZnO	Antibacterial	[44]
*Camellia sinensis*	Plant	ZnO	Antifungal	[45]
Various	Plant Fruit Vegetable residue	ZnO	Antibacterial Antifungal Antioxidant Anti-inflammatory Antidiabetic Anticancer	[46]
*Lupinus albus*	Plant	ZnO	Antibacterial Photocatalytic Activity	[47]
Various	Plant Microorganisms	ZnO	Photocatalytic Activity CO_2_ Conversion	[48]
*Magnolia officinalis* *Goldthread Lonicera japónica*	Plant	ZnO	Antibacterial Antifungal Fruit preservative	[49]
Chemical synthesis	Various reagents	ZnO	Antibacterial Photocatalytic Activity	[51]
*Nostoc* sp.	Bacteria	ZnO	Antibacterial Antibiofilm	[52]
*Gleocapsa gelatinosa*	Cyanobacteria	ZnO	Antibacterial Antibiofilm	[53]
Commercial Nanoparticles	-	ZnO	Antibacterial Synergistic effect	[56]
Chemical synthesis	Zinc acetate	ZnO	Antibacterial Synergistic effect	[57]
*S. epidermidis CECT 4183*	Bacteria	Ag/AgCl ZnO	Antibacterial Antibiofilm	This work

## Data Availability

The data that support the findings of this study are available from the corresponding author upon reasonable request.

## Supplementary Materials

The following supporting information can be downloaded at https://www.mdpi.com/article/10.3390/toxics13060478/s1, Table S1. Experimental design and experimental data obtained for the biosorption of Ag(I) by *Staphylococcus epidermidis* CECT 4183. Table S2. Experimental design and experimental data obtained for the biosorption of Zn(II) by *Staphylococcus epidermidis* CECT 4183. Table S3. Kinetic parameters of Zn(II) biosorption with *Staphylococcus epidermidis* CECT 4183. Table S4. ANOVA for the response surface reduced quadratic model for the biosorption of Ag(I) by *Staphylococcus epidermidis* CECT 4183. Table S5. ANOVA for the response surface reduced quadratic model for the biosorption of Zn(II) by *Staphylococcus epidermidis* CECT 4183.
