# Supplementary material for: Removal of Zn(II) and Ag(I) by Staphylococcus epidermidis CECT 4183 and Biosynthesis of ZnO and Ag/AgCl Nanoparticles for Biocidal Applications
Source: Toxics. 2025 Jun 5;13(6):478. doi: 10.3390/toxics13060478 (PMC12197704; doi:10.3390/toxics13060478)
Supplement: Supplementary file 1 [file toxics-13-00478-s001.zip › toxics-3650484-supplementary.pdf]

Removal of Zn(II) and Ag(I) by *Staphylococcus epidermidis* CECT 4183 and biosynthesis of ZnO and Ag/AgCl nanoparticles for biocidal applications

Suplementary Materials

Table S1. Experimental design and experimental data obtained for the biosorption of Ag(I) by *Staphylococcus epidermidis* CECT 4183.

Run	pH	Biosorbent dose (g/L)	q (mg/g)
1	5.75	0.55	33.96
2	4.50	0.30	32.47
3	5.75	0.55	37.60
4	5.75	0.20	23.90
5	5.75	0.55	36.36
6	7.00	0.80	37.28
7	4.50	0.80	41.68
8	7.00	0.30	15.07
9	5.75	0.55	37.09
10	5.75	0.90	39.49
11	5.75	0.55	36.15
12	7.52	0.55	43.71
13	3.98	0.55	35.60

Table S2. Experimental design and experimental data obtained for the biosorption of Zn(II) by *Staphylococcus epidermidis* CECT 4183

Run	pH	Biosorbent dose (g/L)	q (mg/g)
1	6.18	0.20	44.13
2	5.16	0.50	37.10
3	5.16	0.08	12.50
4	6.63	0.50	36.60
5	5.16	0.50	39.45
6	6.18	0.80	39.06
7	5.16	0.50	38.00
8	5.16	0.50	39.55
9	4.20	0.80	35.38
10	4.20	0.20	41.63
11	5.16	0.90	35.33
12	3.80	0.50	37.75
13	5.16	0.50	38.65

Table S3. Kinetic parameters of Zn(II) biosorption with *Staphylococcus epidermidis* CECT 4183

	Lagergren's model	Ho's model
q_e (mg/g)	70.56	85.49
k_0	3×10^{-4} *	4.3×10^{-6} **
R^2	0.94	0.92
ε (mg/g)	5.81	6.37

* min^{-1}
** g/(mg min)
R^2 = coefficient of determination
ε = standard error of estimate

Table S4. ANOVA for the response surface reduced quadratic model for the biosorption of Ag(I) by *Staphylococcus epidermidis* CECT 4183.

Source	Sum of squares	Degree of freedomF	Mean square	F-value	p-value Prob > F
Model	205.80	3	68.60	131.33	< 0.0001
A: pH	18.12	1	18.12	34.70	0.0020
B: Biosorbent dose (g/L)	177.93	1	177.93	340.61	< 0.0001
B^2	35.05	1	35.05	67.09	0.0004
Residual	2.61	5	0.5224		
Lack of fit	1.27	2	0.6358	1.42	0.2885
Pure error	1.34	3	0.4467		
Cor total	208.42	8			
Std. Dev.	0.7228				
C.V. %	2.02				
R-Squared	0.9875				
Adj R-Squared	0.9799				

Table S5. ANOVA for the response surface reduced quadratic model for the biosorption of Zn(II) by *Staphylococcus epidermidis* CECT 4183.

Source	Sum of squares	Degree of freedomF	Mean square	F-value	p-value Prob > F
Model	65.93	4	16.48	43.37	< 0.0001
A: pH	10.43	1	10.43	27.45	0.0001
B: Biosorbent dose (g/L)	0.0030	1	0.0030	0.0080	0.9300
AB	25.47	1	25.47	67.02	< 0.0001
B^2	5.91	1	5.91	15.55	0.0015
Residual	5.32	14	0.3800		
Lack of fit	0.6470	2	0.3235	0.8307	0.4593
Pure error	4.67	12	0.3895		
Cor total	71.25	18			
Std. Dev.	0.6165				
C.V. %	1.62				
R-Squared	0.9253				
Adj R-Squared	0.9040				
